# Supplementary material for: Real-Time Ultrasonography and the Evaluation of Static Images Yield Different Results in the Assessment of EU-TIRADS Categories
Source: J Clin Med. 2023 Sep 6;12(18):5809. doi: 10.3390/jcm12185809 (PMC10532169; doi:10.3390/jcm12185809)
Supplement: Supplementary file 1 [file jcm-12-05809-s001.zip › jcm-2541176-supplementary.pdf]

Table S1. Percentage of concordant assignments of EU-TIRADS category on static US in relation to real-time US as compared to the number of analyzed images (sections) and the nodule's size for raters A, B and C.

% (No.) of concordant assignments of EU-TIRADS category on static US in relation to real-time US					
**rater A**	**rater B**	**rater C**	**rater A**	**rater B**	**rater C**
**single image - transverse section**			**two images - both sections**		
81.8 (54 out of 66)	71.4 (60 out of 84)	74.1 (109 out of 147)	74.8 (151 out of 202)	74.7 (148 out of 198)	67.6 (98 out of 145)
**nodules ≤ 10 mm**			**nodules > 10 mm**		
74.2 (46 out of 62)	70.7 (29 out of 41)	62.5 (30 out of 48)	77.2 (159 out of 206)	74.3 (179 out of 241)	72.5 (177 out of 244)

No statistically significant differences were observed

Table S2. Frequencies of particular categories of EU-TIRADS and sonographic risk features as assigned by raters A, B and C on static US – analysis of 842 nodules.

category/feature	% (No.) of cases			p
	**rater A**	**rater B**	**rater C**	
**EU-TIRADS 2**	1.1 (9)	1.0 (8)	1.0 (8)	NS
**EU-TIRADS 3**	34.7 (292)	41.9 (353)	41.3 (348)	<0.005 A vs. B & C
**EU-TIRADS 4**	44.7 (376)	42.9 (361)	37.3 (314)	<0.005 C vs. A<br><0.05 C vs. B
**EU-TIRADS 5**	19.6 (165)	14.3 (120)	20.4 (172)	<0.005 B vs. A & C
**marked hypoechogenicity**	6.8 (57)	1.3 (11)	3.3 (28)	<0.005 A vs. C<br><0.0001 A vs. B
**non-oval shape**	12.7 (107)	10.6 (89)	13.2 (111)	NS
**irregular margins**	7.6 (64)	4.6 (39)	5.7 (48)	<0.05 A vs. B
**microcalcifications**	1.3 (11)	0.5 (4)	2.0 (17)	<0.01 B vs. C
**hypoechogenicity**	53.8 (453)	51.8 (436)	48.3 (407)	<0.05 A vs. C
**solid composition**	69.2 (583)	80.9 (682)	91.3 (770)	<0.0001 A vs. B & C
**cystic/spongiform**	1.3 (11)	1.2 (10)	1.1 (9)	NS
**macrocalcifications**	6.9 (58)	10.6 (89)	6.4 (54)	<0.01 B vs. A & C

Table S3. Concordance of nodule classification into EU-TIRADS categories among participating raters evaluating static US in relation to the number of analyzed images as well as nodule's size: nodules ≤ 10 mm or larger.

% (No.) of concordant assignment of EU-TIRADS category – static US							
**single image – transverse section (297)**				**two images – both sections (545)**			
**Rater**	**A**	**B**	**All 3**	**rater**	**A**	**B**	**All 3**
**B**	72.1 (214)	-	62.0 (184)	**B**	71.4 (389)	-	57.8 (315)
**C**	75.4 (224)	74.4 (221)		**C**	70.6 (385)	71.2 (388)	
**nodules ≤ 10 mm (151)**				**nodules >10 mm (691)**			
**Rater**	**A**	**B**	**All 3**	**rater**	**A**	**B**	**All 3**
**B**	66.2 (100)	-	55.0 (83)	**B**	72.8 (503)	-	60.2 (416)
**C**	74.8 (113)	67.5 (102)		**C**	71.8 (496)	73.4 (507)	

No statistically significant differences were observed
